# Readmissions After Distal Radius Fracture Open Reduction and Internal Fixation: An Analysis of 11,124 Patients

**DOI:** 10.5435/JAAOSGlobal-D-20-00110

**Published:** 2020-07-20

**Authors:** Rohil Malpani, Tamara S. John, Michael R. Mercier, Taylor D. Ottesen, Afamefuna M. Nduaguba, Matthew L. Webb, Jonathan N. Grauer

**Affiliations:** From the Department of Orthopaedics and Rehabilitation, Yale School of Medicine, New Haven, CT (Mr. Malpani, Mr. Mercier, Mr. Ottesen, Dr. Nduaguba, and Dr. Grauer), and the Department of Orthopedic Surgery, Perelman School of Medicine University of Pennsylvania, Philadelphia, PA (Dr. John, and Dr. Webb).

## Abstract

**Purpose:**

Distal radius fracture (DRF) open reduction and internal fixation (ORIF) is a common surgical procedure. This study assesses reasons and risk factors for readmission after DRF ORIF using the large sample size and follow-up of the American College of Surgeons National Surgical Quality Improvement Program database.

**Methods:**

Adult patients who underwent DRF ORIF were identified in the 2011 to 2016 National Surgical Quality Improvement Program database. Patient demographics, comorbidity status, hospital metrics, and 30-day perioperative outcomes were tabulated. Readmission, time to readmission, and reason for readmission were assessed. Reasons for readmission were categorized. Risk factors for readmission were assessed with multivariate analyses.

**Results:**

Of 11,124 patients who underwent DRF ORIF, 196 (1.76%) were readmitted within 30 days. Based on multivariate analysis, predictors of readmission (*P* < 0.05) were as follows: American Society of Anesthesiologist class > 3 (Odds ratio [OR] = 2.87), functionally dependent status (OR = 2.25), diabetes with insulin use (OR = 1.97), and staying in hospital after the index surgery (inpatient procedure, OR = 2.04). Readmissions occurred at approximately 14 days postoperatively. Of the recorded reasons for readmission after DRF ORIF, approximately one quarter were for surgical reasons, whereas over 75% of readmissions were for medical reasons unrelated to the surgery.

**Conclusion:**

This study found the rate of 30-day unplanned readmissions after DRF ORIF to be 1.76%. Demographic, comorbid, and perioperative factors predictive of readmission were defined. Most postoperative readmissions were for medical reasons unrelated to the surgical site and occurred at an average of approximately 2 weeks postoperatively. Multivariate analysis found that patients with increased American Society of Anesthesiologist class > 3, functional dependence, insulin-dependent diabetes, and those who underwent inpatient surgery for any reason were at a greater risk for readmission. Understanding these factors may aid in patient counseling and quality improvement initiatives, and this information should be used for risk stratification and risk adjustment of quality measures.

Distal radius fracture (DRF) is one of the most common fractures in adults accounting for up to 18% of all fractures in the elderly age group, and recent data suggest a trend toward increasing incidence.^1^ Open reduction and internal fixation (ORIF) of these injuries is increasing in frequency,^2^ and measures of quality are under increasing scrutiny. One such measure is the rate of postoperative readmissions.^3^ In fact, national programs such as the Hospital Readmissions Reduction Program have been developed under the new Patient Protection and Affordable Care Act to help incentivize system-wide changes that address metrics such as readmission.^4,5,6^ For example, hospitals can now be penalized up to 3% of their reimbursements for failing to meet the established readmission benchmarks.^4,7^

Previous studies that investigated surgical outcomes after DRF ORIF have not focused on readmissions.^8,9,10^ Single institution studies are limited by a small sample size and limited generalizability, Goodman et al^11^ studied 314 ORIFs with one unplanned revision surgery within 30 days of the procedure. Other studies have used state databases, which have the limitations inherent to administrative databases,^12,13,14^ or have grouped hand and forearm diagnoses.^11,15,16,17^ This makes it difficult to interpret the results, with some studies having over 300 procedural codes aggregated in a single analysis.

This study was thus performed to investigate the national rate of readmission after ORIF of DRF and to assess predictive factors, reasons for readmission, and timing of readmissions. To accomplish this goal, we used the large sample size, high data quality, and nationally representative cohort available in the American College of Surgeons National Surgical Quality Improvement Program (NSQIP) database. Our findings could be useful for patient counseling, medical clearance, and postoperative management. These findings will also be useful for readmission benchmarking, risk stratification, and risk adjustment.

## Methods

### Data Source and Patient Population

A retrospective analysis was performed using data from the 2011 to 2016 NSQIP database. This database is a clinical registry which collects over 150 perioperative and demographic variables from over 650 hospitals in the United States.^18^ In recent years, the NSQIP has become widely accepted as a reliable instrument for analyzing adverse event (AE) data associated with orthopaedic surgical procedures.^19,20^

All adult patients aged 18 years and older undergoing DRF ORIF were identified using the following Current Procedural Terminology (CPT) codes: 25607 (open reduction and internal fixation for extra-articular fracture), 25608 (intra-articular fracture), and 25609 (comminuted fracture). Patients with missing sex, height, weight, the American Society of Anesthesiologists (ASAs) classification, or functional status data were excluded, and this comprised less than 1% of the study population. A total of 11,124 patients were analyzed in this study.

This research is exempt from review under the parts of the federal regulation 45 Code of Federal Regulations 46.101(b)(4). This part of the federal regulations covers research involving the collection or study of existing data, documents, and records if these sources are publicly available or if the information is recorded by the investigator in such a manner that subjects cannot be identified. The NSQIP database meets both of these criteria, and studies using this database have been deemed exempt from review by the Institutional Review Board at our institution. The NSQIP database and the hospitals participating in the NSQIP database are the source of the data used herein; they have not verified and are not responsible for the statistical validity of the data analysis or the conclusions derived by the authors.

### Data Elements

Readmission after surgery is reported as a discrete data element in the NSQIP database. Thus, the patient population was separated into 2 groups: those readmitted within 30 days after surgery and those not readmitted within 30 days after surgery.

Patient characteristics compared between the 2 groups included age, sex, and functional status; ASAs classification which were directly abstracted form the data set; and body mass index (BMI) which was calculated from height and weight (weight [kg]/height [m]^2^). Specific comorbidities assessed included diabetes, steroid use for a chronic condition, dyspnea, smoking status, and hypertension (requiring medication).

Surgical characteristics were also compared between the 2 groups. Data elements included CPT codes, anesthesia type (general or regional), surgical time, hospitalization status postoperatively (admitted postoperatively or discharged postoperatively), and discharge location (home or location other than home). Surgical times greater than 3 SDs above the mean were removed from the analysis, and surgical time greater than 110 minutes (1 SD [35 minutes] above the mean [75 minutes]) was classified as prolonged surgical time.

AEs were directly abstracted form the database and were categorized by severity (“major” and “minor”) consistent with previous studies.^21^ AEs classified as “major” included death, sepsis/septic shock, unplanned intubation, ventilator use >48 hours, stroke, cardiac arrest, myocardial infarction, acute renal failure, pulmonary embolism, deep vein thrombosis, wound infection, and return to the operating room. “Minor” AEs included wound dehiscence, urinary tract infection, pneumonia, progressive renal insufficiency, and transfusion. “Any” AEs included occurrence of either a “major” or a “minor” AE.

Reason for readmission was then identified. The NSQIP reports reasons for readmission using the International Classification of Diseases (ICDs) code. These codes were used to categorize reasons for readmission into groups into related to surgical factors and those related to medical factors unrelated to the surgery. Time to readmission is a discrete data element abstracted from the NSQIP. AEs between the date of the original DRF ORIF and readmission were counted.

### Statistical Analysis

Pearson chi-square test was used to analyze demographic, surgical, and comorbidity variables, whereas two-tailed Student *t*-tests was used for continuous variables. Statistical significance was set at a *P*-value of α < 0.05. A multivariable logistic regression was used to determine the independent association of each risk factor with readmission while controlling for all other reported demographic, perioperative, and comorbid factors. Controlled covariates and factors in the analysis included age, sex, BMI, functional status (independent versus dependent), ASA classification (scores 1 and 2, versus 3 and 4), hospitalization status (whether patient remained inpatient versus outpatient postoperatively), anesthesia (general versus regional), surgical time (greater than or less than 110 minutes), discharge location (to home or not), fracture type determined by CPT code, and diabetes with and without insulin-dependence. Statistical analysis was performed in Stata version 13.1 (StataCorp, LP).

## Results

### Patient Population

In total, 11,124 patients who underwent DRF ORIF were identified based on the defined criteria. Among this cohort, 196 (1.76%) were readmitted within 30 days of the procedure.

### Univariate Assessment of Factors Associated with Readmission

Preoperative demographic and comorbidity factors of those readmitted and not readmitted are summarized in Table 1 and Table 2. Compared with the nonreadmitted group, the readmitted group were older (*P* < 0.001), had greater BMI (*P* < 0.001), were less often functionally independent (*P* < 0.001), and had greater ASA class (*P* < 0.001) (Table 1). Furthermore, those who were readmitted had a greater incidence of diabetes (*P* < 0.001), steroid use for chronic conditions (*P* = 0.002), dyspnea (*P* = 0.010), smoking history (within 1 year) (*P* < 0.001), and hypertension (requiring medication) (*P* < 0.001) (Table 2).

**Table 1:** Demographics of Patients Who Underwent Distal Radius Fracture Open Reduction and Internal Fixation, Organized by Readmission Status

Type	Patient not Readmitted	Patient Readmitted	Univariate *P* value
Cases (N = 11,124) (100%)	10,928 (98.24%)	196 (1.76%)	
Age			**<0.001**
18-54	4,145 (37.93%)	47 (23.98%)	
55-64	2,977 (27.24%)	46 (23.47%)	
65-74	2,276 (20.83%)	56 (28.57%)	
≥75	1,530 (14.00%)	47 (23.98%)	
Sex			0.357
Male	2,942 (26.92%)	47 (23.98%)	
Female	7,986 (73.08%)	149 (76.02%)	
BMI (mass [kg]/height [m]2)			**<0.001**
<25	3,986 (36.48%)	63 (32.14%)	
25-30	3,527 (32.27%)	60 (30.61%)	
30-35	2,002 (18.32%)	28 (14.29%)	
>35	1,413 (12.93%)	45 (22.96%)	
Functional status (before surgery):			**<0.001**
Independent	10,737 (98.25%)	179 (91.33%)	
Partially dependent	178 (1.63%)	16 (8.16%)	
Totally dependent	13 (0.12%)	1 (0.51%)	
ASA			**<0.001**
1	2,064 (18.89%)	13 (6.63%)	
2	5,930 (54.26%)	63 (32.14%)	
3	2,768 (25.33%)	110 (56.12%)	
≥ 4	166 (1.52%)	10 (5.10%)	

P-values that were significant (<0.05) are in bold. ASA = American Society of Anesthesiologists classification; BMI, body mass index

**Table 2:** Additional Comorbidities of Patients Who Underwent Distal Radius Fracture Open Reduction and Internal Fixation

Type	Patient not readmitted	Patient readmitted	Univariate *P* value
Cases (N = 11,124) (100%)	10,928 (98.24%)	196 (1.76%)	
Diabetes			**<0.001**
Insulin-dependent diabetes	329 (3.01%)	20 (10.20%)	
Noninsulin-dependent diabetes	606 (5.55%)	20 (10.20%)	
Steroid use for chronic condition			**0.002**
Yes	215 (1.97%)	10 (5.10%)	
No	10,713 (98.03%)	186 (94.90%)	
Dyspnea			**0.010**
None	10,607 (97.06%)	184 (93.88%)	
Moderate exertion	296 (2.71%)	10 (5.10%)	
At rest	25 (0.23%)	2 (1.02%)	
Smoker (within 1 yr)	2,038 (18.65%)	55 (28.06%)	**<0.001**
Hypertension (requiring medication)	3,621 (33.14%)	113 (57.65%)	**<0.001**

P-values that were significant (<0.05) are in bold. Organized by Readmission Status

Surgical characteristics of those who were not readmitted and those who were readmitted are shown in Table 3. Those who were readmitted more often had prolonged operating time (*t*-test *P* = 0.024), inpatient surgery (23.6% versus 49.5%, *P* < 0.001), discharge to a location other than home (3.9% versus 16.8%, *P* < 0.001), major AEs (1.13% versus 36.7%, *P* < 0.001), minor AEs (0.9% versus 12.8%, *P* < 0.001), and any AEs (1.9% versus 44.4%, *P* < 0.001). On univariate analysis, fracture morphology (based on CPT code) and type of anesthesia used (general versus regional) were not associated with being readmitted.

**Table 3:** Surgical Characteristics of Patients Who Underwent DRF Open Reduction and Internal Fixation, Organized by Readmission Status

Type	Patient not Readmitted	Patient Readmitted	Univariate *P* value
Cases (N = 11,124) (100%)	10,928 (98.24%)	196 (1.76%)	
Procedure by CPT code			0.474
ORIF of extra-articular DRF (25607)	4,005 (36.65%)	68 (34.69%)	
ORIF of intra-articular DRF (25608)	3,575 (32.71%)	60 (30.61%)	
ORIF of comminuted DRF (25609)	3,348 (30.64%)	68 (34.69%)	
Anesthesia			0.064
General	8,936 (81.77%)	171 (87.24%)	
Regional	1,994 (18.25%)	25 (12.76%)	
Surgical time			
Mean, minutes (SD)	75.28 (34.97)	81.08 (36.44)	**0.024**
Surgical time > 110 minutes (mean + 1 SD)	1,528 (13.98%)	27 (13.78%)	0.140
Hospitalization status (postoperatively)			**<0.001**
Inpatient (length of stay ≥ 1 ie, admitted post-op)	2,582 (23.63%)	97 (49.49%)	
Outpatient (length of stay = 0 ie, discharged post-op)	8,342 (76.34%)	99 (50.51%)	
Discharge location			**<0.001**
Discharged to home	10,506 (96.14%)	163 (83.16%)	
Discharged to location other than home	422 (3.86%)	33 (16.84%)	
Major adverse events	124 (1.13%)	72 (36.73%)	**<0.001**
Minor adverse events	95 (0.87%)	25 (12.76%)	**<0.001**
Any adverse events	207 (1.89%)	87 (44.39%)	**<0.001**

P-values that were significant (<0.05) are in bold. CPT = Current Procedural Terminology, DRF = distal radius fracture, ORIF = Open Reduction and Internal Fixation

### Multivariable Assessment of Factors Associated with Readmission

**Figure 1:**
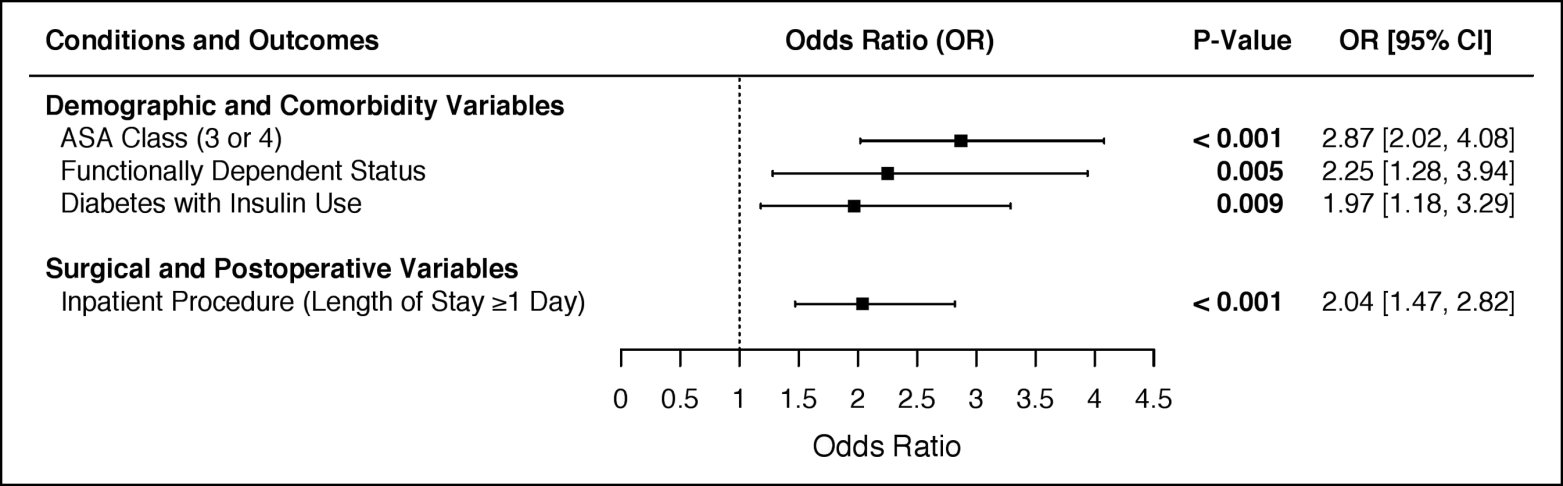
Forest plot depicting the significant variables from the multivariate regression on both demographic, and surgical and post-operative factors highlighting factors impacting readmission following Distal Radius ORIF Procedures. OR=Odds Ratio; CI=Confidence Interval.

In considering demographic, comorbidity, and surgical variables in a logistic multivariable regression, the variables that were significant predictors of readmission after DRF ORIF were ASA class 3 or 4 (odds ratio [OR]: 2.87, *P* < 0.001), functionally dependent status (OR: 2.25, *P* = 0.005), inpatient procedure (OR: 2.04, *P* < 0.001), and insulin-dependent diabetes (OR: 1.97, *P* = 0.009) (Table 4 and Figure 1).

**Table 4:** Multivariate Analysis of Factors Associated With Readmission After Distal Radius Fracture Open Reduction and Internal Fixation

Type	Likelihood of Readmission		
Total Cases (N = 11,124)
	OR	95% CI	*P* value
Demographic and comorbidity variables			
Male	1.04	0.72-1.51	0.833
Age	1.01	1.00-1.02	0.219
BMI	1.00	0.98-1.02	0.849
ASA class of 3 or 4	2.87	2.02-4.08	**<0.001**
Functionally dependent status	2.25	1.28-3.94	**0.005**
Noninsulin-dependent diabetes	1.18	0.708-2.09	0.954
Insulin-dependent diabetes	1.97	1.18-3.29	**0.009**
Surgical and postoperative variables			
Inpatient procedure (length of stay ≥ 1 d)	2.04	1.47-2.82	**<0.001**
Regional anesthesia	0.74	0.48-1.14	0.168
Prolonged surgical time	1.13	0.77-1.67	0.528
Discharged to home	0.67	0.42-1.08	0.099

P-values that were significant (<0.05) are in bold. ASA = American Society of Anesthesiologist, BMI = body mass index, OR = odds ratio

Factors in Model: Age, Sex, BMI, Functional Status (independent versus dependent), ASA, Hospitalization Status, Anesthesia, Operative time, Discharge Location, Fracture Morphology.

Variables that were not notable predictors of readmission were diabetes without insulin use, prolonged operating time (defined as mean + 1 SD), male gender, age, BMI, use of regional anesthesia, and a discharge to home.

### Reasons for Readmission

The reasons for readmission were characterized as either related or unrelated to the surgery for 153 of the 196 readmissions (78.06%) based on the ICD-9/10 codes provided in the NSQIP database. The remaining 43 patients did not have a specific reason for readmission recording in the database.

Of the readmissions after DRF ORIF, 37 were characterized as related to the surgical factors (0.33% of all DRF ORIF, 18.9% of all readmissions, amd 24.2% of all readmissions with a recorded reason for readmission, Table 5). The most common reasons for readmission in this cohort were surgical site infections (12 patients), pain-related (8 patients), and medication overdose (4 patients) etiologies.

**Table 5:** Reasons for Readmission After Distal Radius Fracture Open Reduction and Internal Fixation

Reason	N	Prevalence in Total	Prevalence in Readmitted	Time to Readmission
		Population of 11,124 (%)	Population of 196 (%)	(mean ± SD in Days)
Overall	196	1.76%	100%	13.42 ± 8.77
Surgical factors	**37**	**0.33%**	**18.88%**	**11.92 ± 9.00**
Surgical site infection	12	0.11%	6.12%	13.75 ± 7.62
Pain-related	8	0.07%	4.08%	3.50 ± 4.96
Medication overdose	4	0.04%	2.04%	9.00 ± 4.97
Tendon/ligament injuries	4	0.04%	2.04%	23.00 ± 8.72
Mechanical complications of implant	3	0.03%	1.53%	17.33 ± 3.51
Hemorrhage/hematoma	3	0.03%	1.53%	4.67 ± 6.43
Allergic reaction to orthopedic implant	2	0.02%	1.02%	13.50 ± 9.19
Carpal tunnel syndrome	1	0.01%	0.51%	27.00
Medical factors	**116**	**1.04%**	**59.18%**	**13.78 ± 8.48**
Gastrointestinal	30	0.27%	15.31%	15.38 ± 8.95
Fracture/dislocation unrelated to original surgery	20	0.18%	10.20%	18.15 ± 5.91
Respiratory	13	0.12%	6.63%	11.69 ± 7.24
Psychiatric	6	0.05%	3.06%	10.00 ± 8.22
Electrolyte imbalance	5	0.04%	2.55%	17.60 ± 8.26
Sepsis	4	0.04%	2.04%	13.25 ± 7.50
Urinary tract infection	4	0.04%	2.04%	14.50 ± 8.10
Renal	4	0.04%	2.04%	15.75 ± 12.01
Heart failure	4	0.04%	2.04%	7.25 ± 5.38
Atrial fibrillation	3	0.03%	1.53%	3.67 ± 0.58
Cerebrovascular accident	3	0.03%	1.53%	4.00 ± 2.00
Inguinal hernia	2	0.02%	1.02%	18.00 ± 12.73
Diabetes with ketoacidosis	2	0.02%	1.02%	8.00 ± 5.66
Reasons with single incidence	16	0.14%	8.16%	12.37 ± 9.55
Unknown	**43**	**0.39%**	**21.94%**	**13.83 ± 9.55**

The three main categories of reasons for readmission are in bold.

Of the remaining readmission after DRF ORIF, 116 were characterized as unrelated to surgical factors (classified as “medical factors”) (1.04% of all DRF repairs, 59.2% of all readmissions, and 75.8% of all readmissions with a recorded reason for readmission Table 5). The most common reasons for readmission in this cohort were gastrointestinal (30 patients), unrelated fracture/dislocation (20 patients), and respiratory (13 patients) etiologies.

**Figure 2:**
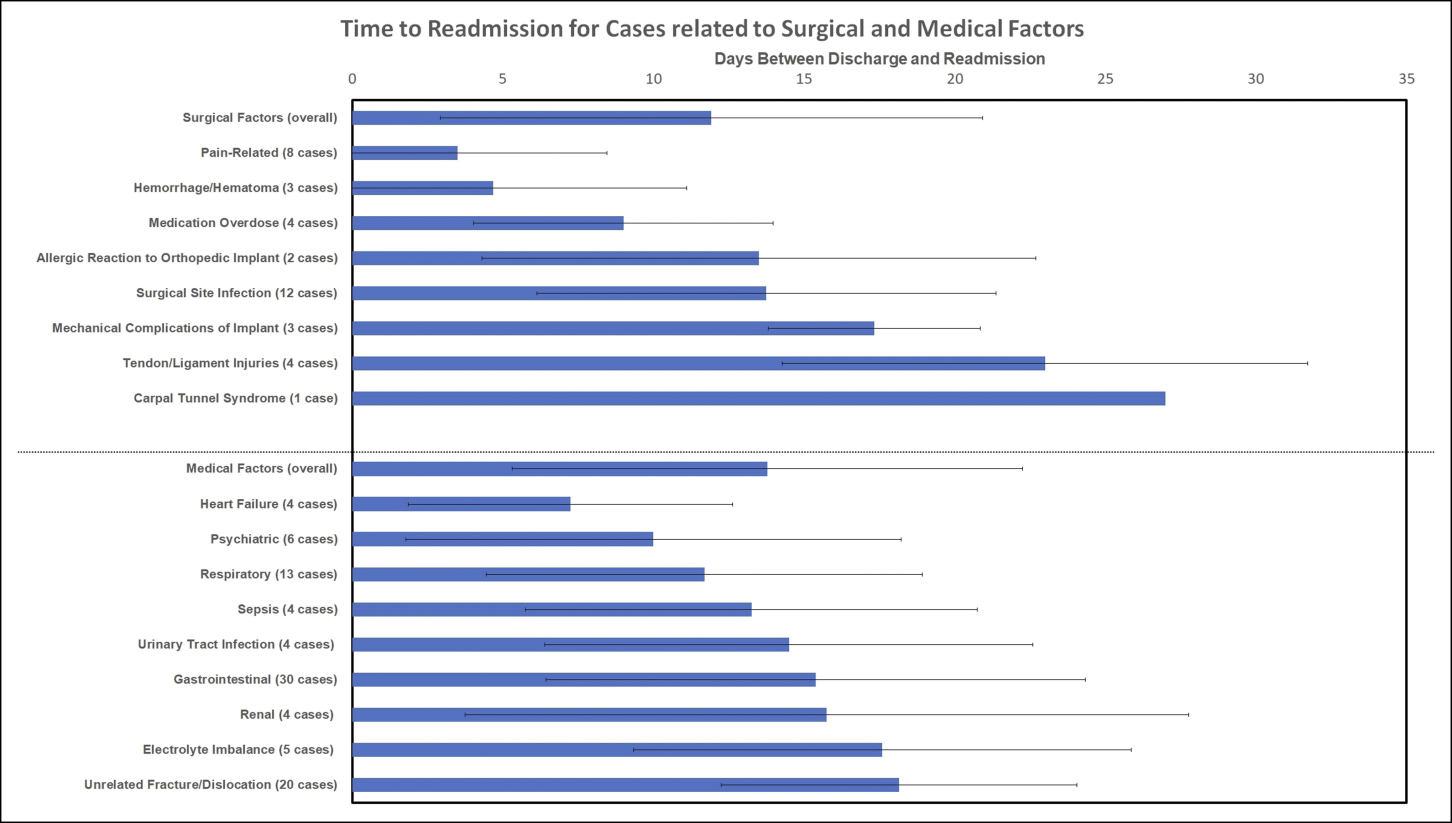
Chart showing time to readmission for cases related to surgical site and unrelated to surgical site. Bar chart depicting the cause of readmission (categorized as related or unrelated to surgical site) on the *y*-axis (along with # of cases for each cause) and mean days between discharge and readmission on the *x*-axis. The SD is depicted with the use of error bars on the bar chart.

The overall average time to readmission in the readmitted cohort was 13.42 ± 8.77 days (Table 5). Time to readmission in the surgical reason cohort was 11.92 ± 9.00 days. Time to readmission in the medical reason unrelated to surgery cohort was 13.78 ± 8.48 days. (Figure 2).

## Discussion

Incidence of DRF ORIF continues to increase.^22^ Thirty-day readmissions are an important quality metric used by the Centers for Medicare & Medicaid Services to track performance and to encourage healthcare institutions to improve the quality of care and to reduce healthcare costs.^23^ The current study used the NSQIP to study unplanned postoperative admissions after DRF ORIF and to categorize risk factors that may predispose patients to readmission.

The rate of unplanned readmissions after DRF ORIF was found to be 1.76%. This is consistent with previous studies that report rates of unplanned readmission after DRF ORIF to be between 0.9% and 8%.^12,17,24^ Based on a multivariable analysis, predictors of readmission after DRF ORIF were identified to be ASA class > 3 (OR = 2.87), functionally dependent status (OR = 2.25), insulin-dependent diabetes (OR = 1.97), and remaining in hospital after the index surgery (inpatient procedure, OR = 2.04).

These identified predictors for readmission correlate with clinical expectations and previous studies of various orthopaedic-related surgeries.^25,26^ Patients with multiple or severe comorbidity would be expected to have greater perioperative needs, require greater postoperative care, and to be at greater risk for readmission.^27,28^ Previous studies have also shown that patients with insulin-dependent diabetes are at greater risk for readmission after total knee arthroplasty and lumbar spine surgery.^29,30^ Patients who underwent inpatient DRF ORIF may be more likely to have conditions that preceded their admission and benefit from an in-hospital multidisciplinary team.^31^ These findings suggest preoperative optimization and close postoperative follow-up may be helpful for these patients.

Compared with other orthopaedic procedures, the rate of readmission after DRF ORIF is low, thus both patients and surgeons do not expect readmissions after surgeries that are commonly considered outpatient. Although the “one-size-fits-all” surgical quality metric of readmission was not conceived for ORIF for DRFs, these quality metrics are currently used, and all surgeons and health systems are accountable to them*.*

Of the patients with known reasons of readmission, readmissions were more likely to be medical reasons unrelated to the surgery (75.8% of readmissions) than reasons related to DRF ORIF surgery (24.2% of readmissions). Of postoperative readmissions related to surgery, the most common reasons were surgical site infection (6.1% of readmissions), poor pain control (4.1% of readmissions), and medication overdose (2.04% of readmissions). The identified rate of surgical site infections is comparable with rates of 1 to 10% reported in the literature.^32,33,34^

Curtin et al^12^ in their review of readmissions after distal radius surgeries reported that 10% of emergency department visits in their patient population were from issues related to postoperative pain and concluded that improved pain control is an easily modified predictor for readmissions. The anesthesia literature highlights the challenges of achieving adequate pain control in the ambulatory setting.^35,36^ Opioid analgesia remains the mainstay of postoperative pain management despite its many known drawbacks that include tolerance, difficulty weaning, and the potential for overdose in an older patient population. Achieving adequate pain control with multimodal anesthesia while minimizing amounts of opioids is difficult. Evolving concepts of achieving adequate anesthesia in outpatient setting include local anesthetic infiltration at the time of surgery and the use of nerve blocks in the postoperative setting. However, one-time infiltration with blocks/local anesthetics while decreasing pain in the immediate period do not having lasting effects.^37^ The current study showed that most readmission from pain occurring within 3 to 4 days after surgery highlighting that this is an early postoperative issue.

Of postoperative readmissions for medical reasons unrelated to the surgery, the most common reasons were gastrointestinal (eg, ileus, gastrointestinal hemorrhage, cholecystitis, 15.3% of readmissions), fracture/dislocation unrelated to original surgery (10.2% of readmissions) and respiratory (eg, pneumonia, acute respiratory failure, 6.6% of readmissions).

Rates of gastrointestinal complications in a general cohort of orthopaedic surgery patients were reported to be 18% in the literature.^28^ Because gastrointestinal causes are of varying etiologies, no single intervention can address prevention of this category. Nonetheless, the importance of limiting postoperative opioid use to minimize postoperative gut dysmotility is noted.^38^ Rates of pulmonary complications causing readmissions reported in the literature in a cohort of patients after outpatient hand and/or elbow surgery was reported to be 3%.^17^ These results highlight the importance of pulmonary optimization and care. Studies that investigate postsurgical respiratory AEs have shown that incentive spirometry and using oral suctioning when appropriate can prevent postoperative pneumonia and other respiratory-related adverse outcomes.^39^

Overall, the mean timing to readmissions was 13.4 ± 8.8 days. This is consistent with a median timing of unplanned readmission reported in the outpatient hand and elbow literature (14 days^17^) and spine/joint arthroplasty literature (12 to 14.5 days^40^).

The current study has several limitations. Although the NSQIP includes data relevant to most surgical patients, it lacks the focus on the outcome measures and complications that are specific to upper extremity patients. In addition, the NSQIP only follows patients for 30 days after surgery so some medical and surgical complications may not be well reported. In addition, an icd-9/10 specified reason for readmission was not available for 21.94% of the cohort. Despite the above-noted limitations, databases such as the NSQIP include high quality, specifically abstracted data, from a large sample size across multiple institutions and this large sample size enables the study of relatively rare occurrences. To mitigate some of these issues, future research could use prospective study design, longer follow-up, and DRF-specific outcomes measures (eg, range of motion, function, and patient-reported outcome measures).

The current study found the rate of 30-day unplanned readmissions after ORIF of DRF to be 1.76%. Demographic, comorbid, and perioperative factors predictive of readmission were defined. Most postoperative readmissions were for medical reasons unrelated to the surgery and occurred at an average of approximately two weeks postoperatively. Understanding these factors may aid in patient counseling and quality improvement initiatives. Furthermore, we found that the independent risk factors for readmission were nonmodifiable patient factors. Patients with increased ASA class, those with insulin-dependent diabetes, those who lacked functional independence, and patients who remained i n hospital for whatever reason were 2 to 3 times more likely to be readmitted after DRF ORIF. This information will be useful for risk stratification and quality measure risk adjustment.
